# Clinical Application of Stem Cell Therapy in Reconstructing Maxillary Cleft Alveolar Bone Defects: A Systematic Review of Randomized Clinical Trials

**DOI:** 10.7759/cureus.23111

**Published:** 2022-03-13

**Authors:** Eman Alfayez, Faisal Alghamdi

**Affiliations:** 1 Oral Biology, Faculty of Dentistry, King Abdulaziz University, Jeddah, SAU

**Keywords:** cleft alveolus, bone regeneration, dental stem cells, stem cells, clinical application

## Abstract

An alveolar cleft is the most common congenital bone defect. This systematic review aimed to investigate the use of stem cells for alveolar cleft repair and summarize the outcomes of clinical research studies. The electronic databases PubMed, Scopus, Web of Sciences, and Google Scholar were utilized to search the literature for relevant studies after administering specific inclusion and exclusion criteria. The search included articles that were published from 2011 to 2021 and specific keywords were used in the databases. The search was completed by two independent reviewers following the Preferred Reporting Items for Systematic Reviews and Meta-Analyses (PRISMA) guidelines.Only four studies satisfied both the inclusion and exclusion criteria and were included in this systematic review. These studies investigated different aspects of bone reconstruction in the maxillary alveolar bone by stem cells, including cell types, clinical applications, biomaterial scaffolds, and follow-up period. The accumulated evidence in this systematic review is limited and insufficient to support the role of stem cell use in bone regeneration of maxillary alveolar bone defects. The outcome of using stem cells was studied only in 57 subjects from the four included studies. Although the noninvasive methods of isolating stem cells make them attractive resources for bone regeneration, more research is required in order to standardize and investigate stem cell therapy. This should be done beforehand in adults in less invasive procedures such as bone defect repair in dentistry prior to considering this type of therapy in this vulnerable patient population.

## Introduction and background

Alveolar cleft reconstruction was first reported in 1901 by Von Eiselberg followed by Lexer in 1908 and Dratcher in 1914 with successful bone grafting attempts in cleft patients [1]. Since then, surgeons have been trying to achieve the best reconstruction outcomes by harvesting and implanting autologous bone in the cleft site at different time points. Primary alveolar bone grafting is performed at an early stage following lip repair [2]. Secondary alveolar bone grafting is usually performed during the mixed dentition before lateral incisor eruption in order to provide bony support for its eruption and stabilization of the maxilla [3].

Cleft patients start their therapeutic journey early in life with multiple maxillofacial reconstruction procedures [4]. Repairing the cleft bone defect requires a bone graft to fill the defect and regenerate the missing bone [4]. Autogenous bone is still considered the preferred graft for alveolar bone reconstruction and the most commonly used one [4-5]. This is due to an abundance of autogenous cells and signaling molecules that encourage healing and induce regeneration in implanted defect sites. However, the limited amount of available bone in pediatric patients and the invasive harvesting procedure have an additional negative impact on cleft patients, with the increased morbidity of having infections, paraesthesia, and scarring [6]. As an alternative, tissue-engineering strategies provide options that can overcome the aforementioned drawbacks by using customized bio-artificial grafts to fill the defect site and regenerate the missing or damaged tissues. Tissue engineering materials that have been used to replace autogenous bone include demineralized bone matrix (DBM), deproteinized bovine bone (DBB), synthetic polymers, and recombinant human bone morphogenetic protein (rhBMP) [7-8]. Then, cells with great growth potential such as stem cells, bioactive molecules, or growth factors can be added to activate the implanted grafts [9]. In cleft alveolus defect studies, stem cells derived from bone marrow, umbilical cord, dental pulp, and human exfoliated deciduous teeth have been isolated and inspected in terms of tissue regeneration [10]. The latter is considered a promising source as cells were harmlessly isolated from naturally exfoliated deciduous teeth pulp [11].

Although numerous experimental studies investigated the use of stem cells in regenerating bone defects in animal models and human clinical trials, few available studies examined the role and potential application of different types of stem cells to repair maxillary alveolar bone defects [12-15]. Only one systematic review was conducted in 2018 [16], they discussed the use of stem cells in bone regeneration and concluded that stem cells were effective in bone tissue repair and regeneration for clinical application in different experimental studies. Thus, the current systematic review aimed to collect, compare, and analyze the outcome of using different types of stem cells in regenerating maxillary alveolar bone defects. Also, it may provide clinicians with various options when selecting stem cells as a bioactive factor loaded within the tissue-engineered scaffold.

## Review

Materials and methods

Two independent reviewers carried out this systematic review in accordance with the Preferred Reporting Items for Systematic Reviews and Meta-Analyses (PRISMA) guidelines [17].

Focus Review Question

The review question was framed as the following:

“Can stem cell therapy be used as a promising future approach in the field of bone reconstruction to treat children and young patients with maxillary alveolar bone cleft defect?”

Information Sources

An electronic search for articles in the English language was performed using PubMed, Scopus, Web of Sciences, and Google Scholar from 2011 to 2021 due to the lack of updated reviews that were covered by this research area in the dental field.

Literature Search Strategy

The literature search strategy was carried out in December 2021 and then updated in February 2022. The search was done by following the PRISMA guidelines using subsequent electronic databases: Public Medline (PubMed), Scopus, Web of Sciences, and Google Scholar. The search was conducted using the following combination of keywords: “cleft alveolus”, “maxilla”, “alveolar bone”, “graft”, “repair”, “stem cells”, “dental pulp”, “dental stem cells”; “human DPSCs”, “SHED”, “MSCs”, “mesenchymal stromal cells”; “deciduous tooth”, “deciduous teeth”, “tooth exfoliation”, “regeneration”, “tissue engineering”, “tissue regeneration”, “bone tissue engineering”, “bone transplantation”, “bone reconstruction” “tissue-engineered bone”, “bone regeneration”, “osteogenesis”, “osteoblast”, “bone substitute”, “scaffold”, and “tissue scaffolds”. A detailed summary of the search strategy can be found in Appendix No. 1.

 Inclusion Criteria

Studies were included if they followed the applied criteria: scientific articles published between 2011 and 2021; scientific articles that were published in the English language; and articles conducted on human subjects only.

Exclusion Criteria

Studies were excluded if they met any of the following applied criteria: review articles; case reports; in vitro/in vivo studies; editorial or personal opinion articles; papers published in a non-English language; papers that illustrated clinical relevance about the regeneration of tissues other than bone; articles that studied stem cells that were not used for cleft alveolus; and articles that discussed the role of stem cells used in bone grafting for the maxillary cleft alveolus by percentages and samples taken from non-human sources.

Critical Appraisal

The reviewers independently assessed the titles and abstracts of the retrieved publications based on the eligibility criteria and PRISMA standards. Disagreements or contradictions between the two reviewers were resolved through discussion and consensus.

Data Extraction

After thoroughly reading the articles and taking into consideration the variables "title, abstract, methods, unilateral/bilateral cleft defects, type of stem cell, type of scaffold material, follow-up period following the surgery, and main results," data were extracted. Both reviewers independently validated the data for completeness and correctness and entered it into standardized Microsoft Office Excel worksheets (Microsoft Corporation, Redmond, WA).

Data Items

Data from the selected studies were gathered and sorted into columns containing the following information: author and year, study design, the number of subjects, age of the patient, unilateral/bilateral cleft defects, type of stem cell, type of scaffold material, follow-up period following the surgery, type of scoring systems/volumetric measurements used, quantity and quality of bone formation measurements, and main outcomes.

Methodological Quality and Risk of Bias Assessment of Included Studies

The methodological quality of each study was performed using the risk of bias assessment tool outlined in the Cochrane Risk of Bias tool - VISualization (robvis) [18]. The Cochrane Collaboration recommends a specific tool to assess the risk of bias in each selected study. The two authors judged the risk of bias of the selected studies based on the following domains: random sequence generation, allocation concealment, blinding of participants and personnel, blinding of outcome assessment, incomplete outcome data, selective reporting, and other sources of bias. Each domain was assessed as “low,” “unclear,” or “high”. These assessments were reported for each selected study in the “risk of bias” figures. The overall risk of bias associated with each study was evaluated as follows: Low risk of bias: all domains were assessed as “low risk”; Unclear risk of bias: at least one domain was assessed as “unclear risk”; and High risk of bias: at least one domain was assessed as “high risk”. The risk of bias was assessed during the process of data extraction, which could influence the outcome of each selected study. The Cochrane Risk of Bias tool - VISualization (robvis) was used to assess bias present in chosen studies and identify papers with intrinsic methodological and design flaws [18].

Types of Outcome Measurements

Primary outcomes: The status of the bone defect at the end of the alveolar cleft bone repair would either be significant new bone formation by stem cell therapies or failure.

Secondary outcomes: The quality and quantity of the newly formed bone in the maxillary alveolar cleft defect by using different scoring systems and volumetric measurements.

Synthesis of Results

Two tables were developed to describe a variety of relevant data. The first table was prepared to include the study characteristics of each included study and the second table included the outcomes of bone formation by using different scoring systems and volumetric measurements.

Statistical Analysis

Meta-analysis was not possible due to the heterogeneity of the included studies. As a result, only parametric data relating to the age of the patients in the included studies are presented as a mean and standard deviation (M ± SD), as well as a descriptive evaluation of the findings.

Results

Study Selection

**Figure 1:**
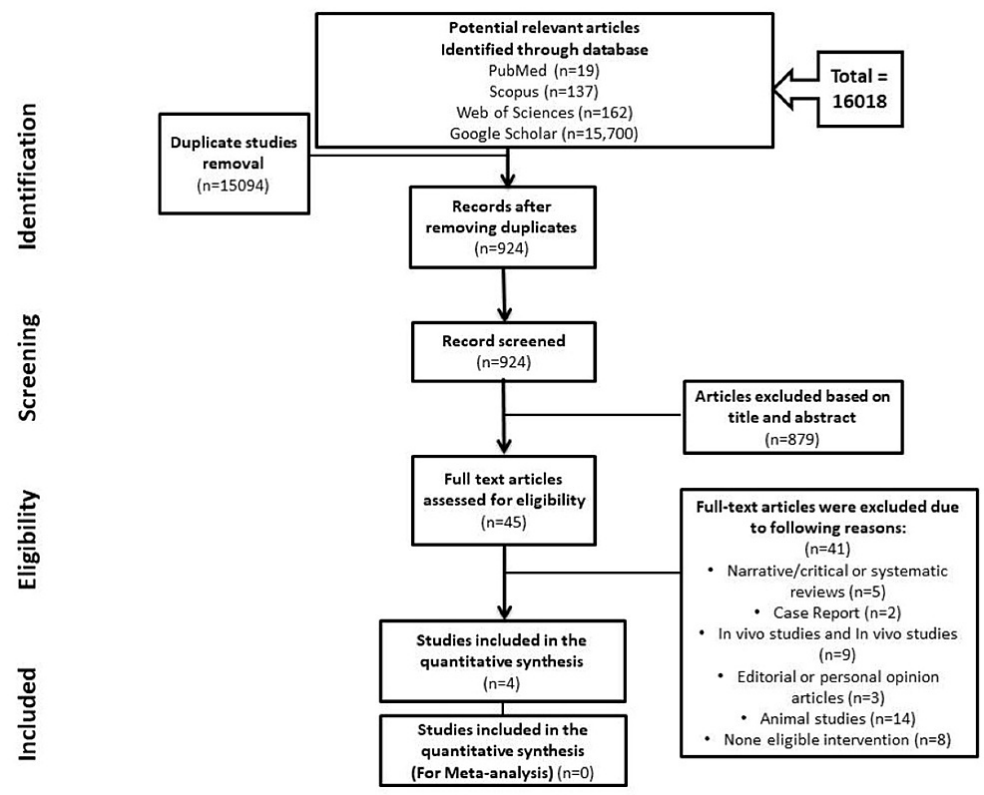
Preferred Reporting Items for Systematic Reviews and Meta-Analyses (PRISMA) flowchart for study selection

Initially, keywords were used to get a total of 16018 articles from databases. A total of 15094 articles were excluded due to title and abstract duplicity or irrelevance. Following assessment for eligibility, only four papers were involved in this review. Figure 1 depicts a summary of the search flow chart for this systematic review.

Study Characteristics

Four human studies that met the inclusion criteria and were conducted during the previous 10 years were included in the search. These studies evaluated the effectiveness of stem cell application in bone regeneration within the maxillary cleft alveolar bone defect. This systematic review included four studies with a total sample of 57 subjects [19-22]. The age of the patients ranged from five months to 10 years in these studies [19-22]. Two studies reported the age of patients with a mean age and standard deviation (mean ± SD) [19,21] while the age of patients was not reported in two studies [20,22] in which one represented patients receiving implants [22]. All types of studies included in this systematic review were randomized clinical trials [19-22]. The stem cell types used included: bone marrow mesenchymal stem cells (BMMSCs) [20,22], umbilical cord stem cells (UCSCs) [21], and deciduous dental pulp stem cells (DDPSCs) [19].

**Figure 2:**
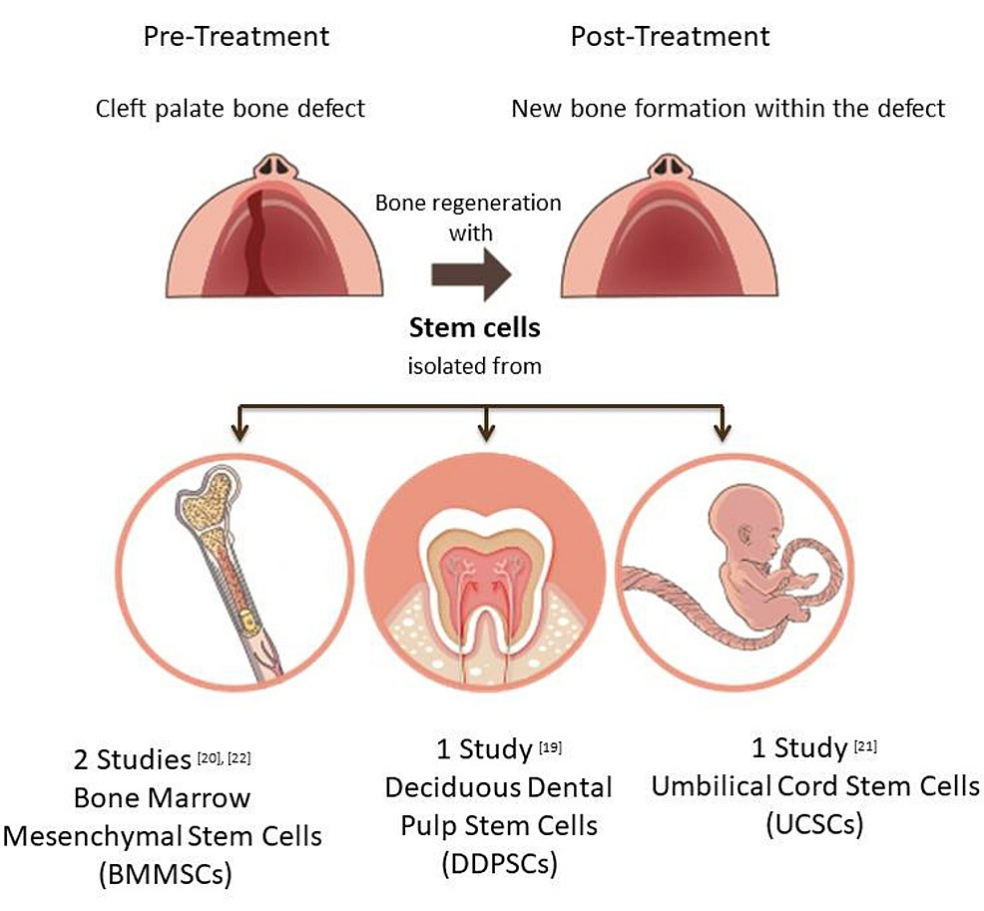
Sources of stem cells used in alveolar cleft defect reconstruction The numbers refer to the number of included studies in this systematic review and summarized in Table 1. BMSCs: Bone marrow stem cells, DDPSCs: Deciduous dental pulp stem cell of human healthy extracted deciduous teeth, UCSCs: Umbilical cord stem cells Source: Refs. [19-22].

Scaffolds were used to support and seed stem cells for bone tissue engineering in the bone defect sites. The different types of scaffolds used in the included studies were summarized in Table 1. Studies showed that extracting autologous stem cells from different tissue types is safe and results in favorable outcomes presented clinically by supporting alveolar bone cleft defects regeneration [19-20,22]. In addition, studies emphasized the importance of using scaffolds and membranes that have osteoinductive and osteoconductive properties to enhance the regeneration capacity of stem cells in the bone defect sites. For example, the use of platelet-rich fibrin (PRF) with BMSCs showed superior outcomes compared to an autogenous iliac crest bone graft [20]. Regarding the cleft defects in this systematic review, three studies included unilateral cleft defects [19-20,22] while one study involved both unilateral and bilateral cleft defects [21]. Regarding the follow-up period following the surgery; studies reported different follow-up periods, including five years in one study [19], 10 years in one study [21], four months in one study [22], and one study did not report the follow-up period [20]. In this section, an informative summary of all included studies and their features is provided in Table 1. A summary of the different types of stem cells used in the included studies for maxillary alveolar bone cleft reconstruction is illustrated in Figure 2.

**Table 1:** Summary of all included studies in this systematic review

Authors	Year	Study Design	Number of Subjects / Age of Patients (Mean ± SD)	Unilateral / Bilateral Cleft Defects	Type of Stem Cells Used	Type of Scaffold Material Used	Follow-Up Period Following the Surgery
Tanikawa DYS, et al. [19]	2020	(Randomized controlled clinical trial)	(n=6) / 10 ± 1.41 years old	Unilateral cleft defects	“Deciduous dental pulp stem cell” (DDPSC)	hydroxyapatite-collagen sponge (250 mg, Geistlich Biomaterials AG, Wolhusen, Germany)	(5 years follow-up)
Mossaad A, et al. [20]	2019	(Randomized controlled clinical trial)	(n=24) / Not Reported	Unilateral cleft defects	“Bone marrow mesenchymal stem cells” (BMMSCs)	Group A: Autogenous iliac crest bone + Group B: Nano calcium hydroxyapatite with a collagen membrane + Group C: Bone marrow stem cells extract and Platelet-rich fibrin (PRF) membrane.	Not Reported
Mazzetti MPV, et al. [21]	2018	(Randomized clinical trial)	(n=9) / 5.11 ± 0.60 months newborns	Unilateral + Bilateral cleft defects	“stem cells from umbilical cord blood and placenta blood”	Autologous stem cells	(10 years follow-up)
Bajestan MN, et al. [22]	2017	(Randomized controlled clinical trial)	(n=18) / Not Reported (Patients receiving implants)	Unilateral cleft defects	Autologous “Bone marrow mesenchymal Stem cells” (BMMSCs)	2 groups: 1. Control group (n=8) 2. Stem cell therapy (n=10): beta-tricalcium phosphate (β-TCP)	(4 months follow-up)

Primary Outcomes

The primary outcomes demonstrated regeneration of the alveolar cleft defect with bone formation following stem cell therapy. Three studies reported neo-bone formation with stem cell application in maxillary alveolar reconstructions [19-20,22]. All these three studies were controlled clinical trials that showed significant bone formation compared to control groups [19-20,22]. Only one study showed a non-significant outcome, in which stem cells were injected into the bone defect without using any scaffold or membrane [21]. An informative description of all included studies and their bone formation outcomes are summarized in Table 2.

**Table 2:** Outcomes of quality and quantity of bone formation and their measurements in this systematic review CBCT: cone-beam computed tomography, CT: computed tomography, HU: Housefield unit, SD: standard deviation, rhBMP-2: recombinant human bone morphogenetic protein-2, BMSCs: bone marrow mesenchymal stem cells, DDPSCs: deciduous dental pulp stem cells

Authors	Year	Type of scoring systems/volumetric measurements used	Quality and quantity of bone formation measurements	Main Outcomes of stem cell therapy
Tanikawa DYS, et al. [19]	2020	- Volumetric analysis of CT images. - 6 and 12 months’ time points. - Superimposition of the images on anatomical landmarks included the pyriform aperture superiorly, and the cement-enamel junction inferiorly.	-The defect at the 6-month follow-up was smaller in the stem cells group (253.2 mm^3^, SD 85.8) and group two (iliac crest bone graft) (260.4 mm^3^, SD 98.5) compared to group one (rhBMP) (393.6 mm^3^, SD 144.7, P=0.048) - At the 12-month follow-up examination, the mean postoperative defect became similar in all groups. - Bone filling percentage at 6-month follow-up was significantly higher with DDPSCs (75.6%, SD 4.8) but at the 12-month follow-up examination, this difference disappeared.	Significant results of bone regeneration compared with traditional iliac crest bone grafting and rhBMP-2.
Mossaad A, et al. [20]	2019	Bone density measurement at the graft site from CT compared to normal side in Housefield unit (HU).	Bone density was higher in the BMSCs group (mean ± SD 618 ± 60.2) compared to the normal side (mean ± SD 375.6 ± 67.9), followed by nano calcium hydroxyapatite with collagen membrane group (mean ± SD 539.9 ± 84.5) compared to normal side with (mean ± SD 395.3 ± 65.9) - The autogenous iliac crest group (mean ± SD 461.0 ± 66.3) compared to normal side (mean ± SD 368.5 ± 68.3) showed resorption in some cases and gave the least values.	Superior bone regeneration with bone marrow stem cells followed by nano calcium hydroxyapatite, both groups showed significant differences compared to the autogenous iliac crest group.
Mazzetti MPV, et al. [21]	2018	Facial tomography in one patient, 2 years postoperatively.	Not reported	There was no evidence of neo-bone formation in cases injected with stem cells.
Bajestan MN, et al. [22]	2017	Ridge width at re-entry was assessed clinically with open bone measurements and radiographically with CBCT.	Bone width was 1.5 ± 1.5 mm in the stem cell therapy group and 3.3 ± 1.4 mm in the control group.	Significant bone formation but there is limited osseous regeneration in large defects.

Secondary Outcomes

The secondary outcomes reported different scoring systems and volumetric measurements to evaluate the quality and quantity of new bone formation postoperatively as shown in Table 2. Three studies evaluated bone formation postoperatively by using computed tomography (CT) scans [19-20]. While one study used a facial tomography scan [21], another one used cone-beam computed tomography (CBCT) [22]. Regarding the quality and quantity of bone formation outcomes, three studies reported positive outcomes with stem cell therapy compared to controls and other test groups [19-20,22]. The measurements of the new bone formation in the defect sites were not reported in one study [21].

Quality and Risk Assessment of the Included Studies

**Figure 3:**
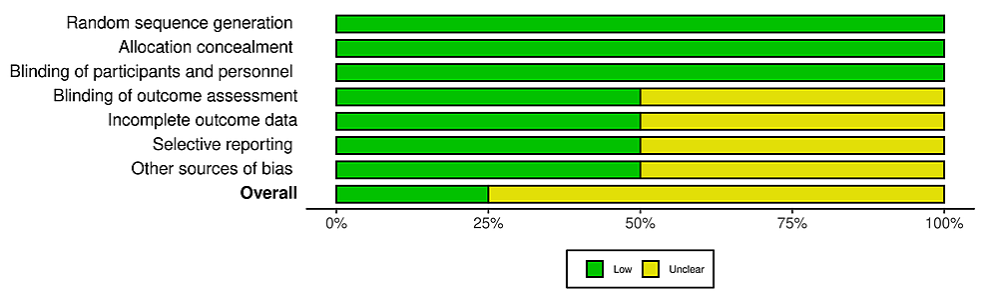
Overall risk of bias summary of all selected studies

**Figure 4:**
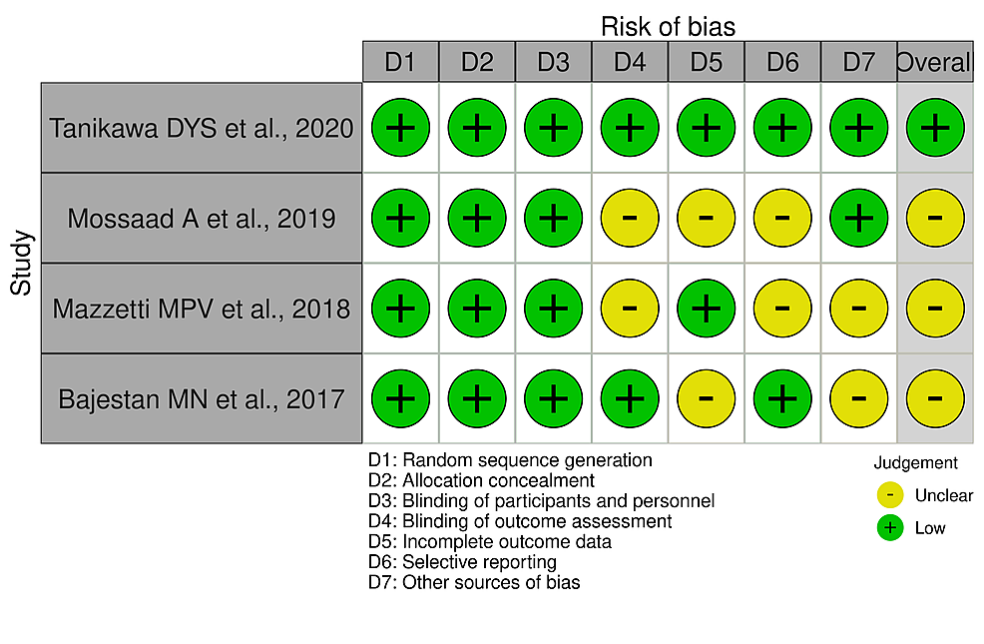
Risk of bias tool of the selected studies (VISualization - (robvis)) Source: Refs. [19-22]

The quality and risk assessment of all included studies were completed by two authors. Included studies were determined by following the Cochrane Risk of Bias tool - VISualization (robvis) [18] to determine the risk of bias. The majority of the included articles had a low risk of bias in the following domains: blinding of outcomes assessment (50%), incomplete outcome data (50%), selective reporting (50%), other sources of bias (50%). All articles demonstrated a low risk of bias (100%) in random sequence generation; allocation concealment; and blinding of participants and personnel domains (Figure 3). Overall, among the four studies, one study (25%) was found to have a low risk of bias [19] and three studies (75%) had an unclear risk of bias [20-22] as shown in Figure 4. The scoring of unclear risk of bias was given to three studies due to lack of sufficient information to make a clear judgment in the following domains: blinding of outcomes assessment, incomplete outcome data, selective reporting, and other sources of bias (Figure 4).

Discussion

This systematic review was conducted to describe and evaluate all research findings in the previous 10 years that satisfied our research objective. It included all the latest clinical studies on the role and application of stem cells for bone reconstruction in maxillary alveolar bone cleft defects. Our review demonstrates a comprehensive set of evidence extracted from four articles that fulfilled our inclusion and exclusion criteria.

Up to date, there is only one recent systematic review that investigated the use of stem cells in clinical application for bone regeneration in bone defects covering the period from 1984 to 2017 and is summarized in Table 3 [16]. Fifty-six studies supported the role of human exfoliated deciduous teeth (SHEDs) and human dental pulp stem cells (hDPSCs) in repairing bone defects, including cranial/calvarial, mandibular, tibial bone, and femoral bone defects [16]. The included studies involved animal experimental models and four human studies, in which three human studies investigated repairing post third molar extraction defects with collagen sponge scaffold and one human clinical trial studied periodontal bone defect regeneration with beta-tricalcium phosphate (β-TCP) scaffold [23-26]. It was concluded that the majority of retrieved studies suggested that stem cells isolated from SHEDs and hDPSCs were effective in bone tissue repair and regeneration for clinical application in animal models or humans [16]. However, alveolar cleft defects were not included in their studies, which warranted the necessity of conducting this review. On the other hand, the only systematic review that investigated tissue engineering strategies for alveolar cleft reconstruction in humans up to the year 2012 included only one stem cell study [27]. However, neither the bone quantity nor quality data were provided [28]. In agreement with previously published systematic reviews, few studies used stem cells in bone regeneration in humans. Most of the included studies in our systematic review favored the use of stem cells in bone regeneration within the alveolar bone defects for cleft patients (Table 1). They used various techniques in extracting, isolating, culturing, and characterizing stem cells. Interestingly, none of the studies reported neither adverse nor negative effects on the clinical application of selected stem cells (Table 1).

**Table 3:** Summary of the recent systematic review included in this systematic review SHEDs: human exfoliated deciduous teeth, hDPSCs: human dental pulp stem cells

Authors	Year	Number of studies using	Method summary	Main Conclusions
Leyendecker Junior A, et al. [16]	2018	56 studies	The systematic review summarises and presents in vivo studies performed from 1984 to November 2017. Using two different databases (PubMed/MEDLINE and Web of Science databases), an electronic search was done.	The use of SHEDs and hDPSCs appears to be effective for bone repair/regeneration as clinical applications for the cleft alveolus.

The use of MSCs with a PRF membrane showed favorable bone regeneration represented in increased bone width and density (Table 2) [20]. CT measurements showed that bone density in the MSCs group was higher (mean ± SD 618 ± 60.2 compared to normal side mean ± SD 375.6 ± 67.9) followed by the nano calcium hydroxyapatite with collagen membrane group (mean ± SD 539.9 ± 84.5 compared to normal side with mean ± SD 395.3 ± 65.9) [20]. Whilst the autogenous iliac crest group resulted in resorption in some cases and gave the least values (mean ± SD 461.0 ± 66.3 compared to normal side mean ± SD 368.5 ± 68.3) [20]. However, the use of MSCs without a scaffold or membrane showed limited osseous regeneration [22]. The same outcome was observed in umbilical cord stem cells (UCSCs) studies; adequate alveolar height when cells were seeded on a gelfoam scaffold while no evidence of neo-bone formation was detected by injecting UCSCs without scaffold or membrane [21]. However, no measurements were reported in this study [21].

The use of DDPSCs in the Tanikawa et al. study not only showed that stem cells harvested from shedding teeth present a reliable source of stem cells for bone regeneration in cleft defects, but it also demonstrated that the numbers of cells harvested from each tooth are sufficient to seed regenerating scaffolds [19]. In addition, the DDPSCs group showed comparable defect size regeneration to the traditional iliac crest graft group and superior bone filling percentage at the six months follow-up (Table 2). However, at the 12-month follow-up, both groups and the rhBMP-2 group showed the same outcome. Although autologous bone graft is considered the gold standard in bone regeneration, it involves second surgical site morbidity with a limited amount of bone to be harvested. In addition to the lengthy operative time and stay in the hospital, there is a risk of intraoperative blood loss, postoperative pain, and high cost [19]. On the other hand, rhBMP-2 adverse effects involved severe swelling in maxillofacial surgery and postoperative nasal stenosis in cleft children [29-30]. As a result, the Food and Drug Administration (FDA) warning was issued against the utilization of it in the pediatric population due to a lack of evidence that confirms long-term effectiveness or safety in children [19].

The use of stem cells isolated from shedding teeth could transform bone regeneration procedures in cleft alveolus cases. This is due to the enrichment of deciduous teeth with stem cells and the ease of isolating them compared to other sources in the human body [19]. Although most of the included studies in this review favor bone marrow stem cells in cleft alveolar bone regeneration in terms of clinical, radiological, and histopathological outcomes, the level of evidence remains low since there are few human studies. This warrants the development of standardized protocols to retrieve and process the different types of stem cells, standardize and report the timing of intervention, the dimension of the maxillary alveolar bone cleft defect to be repaired, and the method of radiological assessment procedure used during follow up stages after surgery. The three-dimensional radiographic evaluation with CT is still considered the most reliable method for the analysis of height and volume of the alveolar bone [15].

In summary, the current review demonstrates an overview of different studies showing the outcomes after using BMMSCs, UCSCs, and DDPSCs for bone regeneration in maxillary alveolar cleft defects (Table 2). It can be concluded that there are insufficient data to support the use of stem cells in alveolar bone cleft repair for now. However, the favorable results from the included studies in this review encourage the need for more clinical trials to be able to consider stem cells as one of the treatment approaches in dentofacial bone regeneration defects. Moreover, standardization and thorough clinical evaluation in adult patients is a prerequisite before investigating this form of therapy in pediatric patients.

Study Strengths and Limitations of This Systematic Review

Our review compiled and evaluated all peer-reviewed publications published in the previous 10 years that met our inclusion criteria. This is the only systematic review that has thoroughly explored the subject of using stem cells in bone regeneration for an alveolar cleft in depth. The systematic review conducted by Leyendecker Junior A et al. (2018) covered the period between 1984 and 2017 [16]. In addition, we used Public Medline [PubMed], Scopus, Web of Sciences, and Google Scholar as search engines. One advantage of using Google Scholar is that it prevents researchers from missing any valuable research that has been published in journals but has not yet been cited in PubMed, Scopus, or Web of Science. On the other hand, we were limited to perform a systematic review without meta-analysis on the selected articles because of the heterogeneity of the confounding factors in the currently involved human studies. In addition, only a few studies were conducted in human subjects that were part of the inclusion criteria and used stem cells in bone reconstruction to treat children and young patients with alveolar clefts.

## Conclusions

This systematic review revealed that there were limited studies on humans using stem cells for alveolar cleft defect repair. They reported encouraging results from stem cell therapy, including significant bone formation in defect sites. A needle aspirate was used to harvest stem cells from bone marrow, and stem cells from extracted or exfoliated teeth were isolated from the dental pulp. Compared to the gold standard bone grafting procedure, which involves donor site morbidity, these noninvasive methods of isolating stem cells make them attractive options for bone repair in the near future. However, more research is needed before the FDA can set suitable standards and restrictions for employing stem cells in alveolar cleft clinical trials. In future clinical studies, it will be critical to standardize a methodology for extracting and processing stem cells in sufficient numbers to enhance bone regeneration within the implanted scaffold.

## Appendices

**Table 4:** Search strategy in the databases for this systematic review

Database	Search Strategy	Results
PubMed From inception up to February 14, 2022 All fields With no limits	#1((cleft alveolus) OR (maxilla)) OR (alveolar bone) #2 (graft) OR (repair) #3 (((((((((stem cells) OR (dental pulp)) OR (dental stem cells)) OR (human DPSCs)) OR (SHED)) OR (MSCs)) OR (mesenchymal stromal cells)) AND (deciduous tooth)) OR (deciduous teeth)) OR (tooth exfoliation) #4 ((((((((((((tissue engineering) OR (tissue regeneration)) OR (regeneration)) AND (bone tissue engineering)) OR (tissue-engineered bone)) OR (bone regeneration)) OR (bone transplantation)) OR (bone reconstruction)) OR (osteogenesis)) OR (osteoblast)) OR (bone substitute)) AND (scaffold)) OR (tissue scaffolds) #5 #1 AND #2 AND #3 AND #4	#5 = 19
Scopus From inception up to February 14, 2022 Title Abstract Key Word With no limits	#1 “cleft alveolus” OR “maxilla” OR “alveolar bone” #2 “graft” OR “repair” #3 “stem cells” OR “dental pulp” OR “dental stem cells” OR “human DPSCs” OR “SHED” OR “MSCs” OR “mesenchymal stromal cells” AND “deciduous tooth” OR “deciduous teeth” OR “tooth exfoliation” #4 “tissue engineering” OR “tissue regeneration” OR “regeneration” AND “bone tissue engineering” OR “tissue-engineered bone” OR”bone regeneration” OR “bone transplantation” OR “bone reconstruction” OR “osteogenesis” OR “osteoblast” OR “bone substitute” AND “scaffold” OR “tissue scaffolds” #5 #1 AND #2 AND #3 AND #4	#5 = 137
Web of Science From inception up to February 14, 2022 TS - Topic With no limits	#1 TS= (“cleft alveolus” OR “maxilla” OR “alveolar bone”) #2 TS= (“graft” OR “repair”) #3 TS= (“stem cells” OR “dental pulp” OR “dental stem cells” OR “human DPSCs” OR “SHED” OR “MSCs” OR “mesenchymal stromal cells” AND “deciduous tooth” OR “deciduous teeth” OR “tooth exfoliation”) #4 TS= (“tissue engineering” OR “tissue regeneration” OR “regeneration” AND “bone tissue engineering” OR “tissue-engineered bone” OR”bone regeneration” OR “bone transplantation” OR “bone reconstruction” OR “osteogenesis” OR “osteoblast” OR “bone substitute” AND “scaffold” OR “tissue scaffolds”) #5 #1 AND #2 AND #3 AND #4	#5 = 162
Google Scholar From inception up to February 14, 2022 TX – All Text With no limits	“cleft alveolus” OR “maxilla” OR “alveolar bone” AND “graft” OR “repair” AND “stem cells” OR “dental pulp” OR “dental stem cells” OR “human DPSCs” OR “SHED” OR “MSCs” OR “mesenchymal stromal cells” AND “deciduous tooth” OR “deciduous teeth” OR “tooth exfoliation” AND “tissue engineering” OR “tissue regeneration” OR “regeneration” AND “bone tissue engineering” OR “tissue-engineered bone” OR “bone regeneration” OR “bone transplantation” OR “bone reconstruction” OR “osteogenesis” OR “osteoblast” OR “bone substitute” AND “scaffold” OR “tissue scaffolds”	15700
